# Patterns and Predictors of Recurrence After Curative Resection of Colorectal Liver Metastasis (CRLM)

**DOI:** 10.1007/s12029-024-01105-8

**Published:** 2024-08-22

**Authors:** Satya Niharika Vadisetti, Mufaddal Kazi, Shraddha Patkar, Rohit Mundhada, Ashwin Desouza, Avanish Saklani, Mahesh Goel

**Affiliations:** 1 Department of Surgical Oncology, Tata Memorial Centre, Homi Bhabha National Institute, Mumbai, Maharashtra India; 2 Division of Colorectal Surgery, Department of Surgical Oncology, Tata Memorial Centre, Homi Bhabha National Institute, Mumbai, Maharashtra India; 3 Division of Hepato-Biliary Surgery, Department of Surgical Oncology, Tata Memorial Centre, Homi Bhabha National Institute, Parel, Mumbai, 400012 Maharashtra India

**Keywords:** Colorectal cancer, Liver metastasis, Liver resection, Recurrence

## Abstract

**Background:**

Our study aims to determine the predictors and patterns of relapses after curative colorectal liver metastasis (CRLM) resection.

**Methods:**

A single-centre, retrospective study of CRLM patients operated between 2010 and 2022 was performed. The site of first recurrence was either hepatic (marginal (≤ 1 cm) or extramarginal), extrahepatic, or both. Factors that predicted relapse patterns and overall survival were determined by multivariable Cox regression analysis with backward elimination of variables.

**Results:**

The study consisted of 258 patients, with a similar proportion of synchronous (144; 56%) and metachronous(114; 43%) metastasis. At a 43-month median follow-up, 156 patients (60.4%) developed recurrences with 33 (21.1%) in the liver, 62(24.03%) extra-hepatic recurrences, and 58 (22.48%) having both. Isolated marginal liver relapses were seen in seven (9.89%) liver recurrence patients. The median overall and relapse-free survivals were 38 months (30–54) and 13 months (11–16), respectively. The 3-year liver-relapse-free survival was 54.4% (44.9–60.6). Size of liver metastases > 5 cm (HR 2.06 (1.34–3.17), involved surgical margins (HR 2.16 (1.27–3.68)), and adjuvant chemotherapy (HR 1.89 (1.07–3.35)) were predictors of hepatic recurrences. Node positivity of primary (HR 1.61 (1.02–2.56)), presence of baseline extra-hepatic metastases (HR 0.30 (0.18–0.51)), size of liver metastases > 5 cm (HR 2.02 (1.37–2.99)), poorly differentiated histology (HR 2.25 (1.28–3.49)), presence of LVI (HR 2.25 (1.28–3.94)), and adjuvant chemotherapy (HR 2.15 (1.28–3.61)) were predictors of extra-hepatic recurrences.

**Conclusion:**

The study found majority relapses occurred at extrahepatic sites whilst isolated marginal recurrences were few. The consistent predictors of recurrence were size and inability to deliver adjuvant therapy. A tailored adjuvant therapy might improve outcomes after liver metastasectomy in colorectal cancers.

## Introduction

The multitude of perioperative treatment options available for resectable colorectal liver metastasis (CRLM) has reduced the probability of recurrences thus providing a high chance of cure. With the increasing safety of liver resection and advances in systemic therapy, various studies now report the 5- and 10-year survival following CRLM resection as 50% and 25%, respectively [1–4]. Despite the improvement in survival, 70% of the resected CRLM recur and 20% of these occur within the first two years within the remnant liver [5, 6]. Disease-free interval after liver resection and the patterns of recurrence have been shown to correlate with long-term prognosis [7, 8]. Location and pathology of primary tumour, R1 margin status, pre-operative CEA levels, extra-hepatic metastasis and adjuvant therapy were few of the factors attributed to recurrence [9–12]. This phenomenon of disease recurrence of after surgical resection of radiological and intra-operative disease is credited to the presence of occult micrometastasis. Planning peri-operative systemic therapy based on molecular profiling has shown to provide significant clinical benefit and is commonly used in initially unresectable CRLM [13, 14]. There is now growing body of evidence for re-resection and local ablative therapies to treat hepatic recurrences where perioperative systemic therapy has limited effect [15, 16].

The knowledge of susceptibility to liver or extrahepatic recurrences occur with the current management will help to improve outcomes by allowing us to tailor post-resection surveillance and treatment strategies. In this background, the systemic therapy could be utilised only for patients at high risk of extrahepatic recurrences whilst the intention of augmenting local therapy with aggressive surgical resection with or without the addition of hepatic artery infusion would be applicable in patients who have a higher propensity for liver relapse. Thus, our study aims to determine these patterns and predictors of relapses after curative CRLM resection to guide the optimum strategy for perioperative therapy.

## Methodology

### Study Design and Setting

A single-centre, retrospective study of patients treated by our hepato-biliary and colorectal disease management units at Tata Memorial Hospital (Mumbai, India). Data was collected from a prospectively maintained database and electronic medical records.

### Patients

Patients of colorectal cancer with synchronous or metachronous liver metastasis who were treated with a curative liver resection between 2010 and 2022 were analysed.

The inclusion criteria were as follows:

1. Patients who were operable and underwent liver resection for CRLM with or without liver-directed therapies
2. Patients with limited extra-hepatic metastasis in the lung and peritoneum at primary presentation that were considered resectable
3. Pathologically confirmed diagnosis of CRLM

Patients with other histologies and those deemed unresectable were excluded.

### Management Strategy

The primary staging modality for colorectal liver metastasis was a contrast-enhanced triple-phase computerised tomography (CECT). Magnetic Resonance Imaging (MRI) and Positron Emission Tomographies (PET) were used selectively for problem-solving in doubtful lesions as per the decision of the multi-disciplinary team (MDT). The decision for perioperative chemotherapy was also an individualised decision based on the location of the primary tumour (colon vs rectum), timing of liver metastasis (synchronous vs metachronous), disease-free interval, and burden of metastatic disease and if the primary was symptomatic. Colorectal liver metastasis were considered synchronous if they presented at the time or within 6 months of primary diagnosis [17]. Similarly, the timing of resection of liver metastasis (simultaneous vs staged) was also based on MDT decisions. When staged resections were performed, a liver-first approach was preferred. No planned positive or R1vascular resections were performed. Adjuvant chemotherapy was considered for all patients who underwent liver resection. However, the receipt of adjuvant therapy depended on complications following surgery, performance status of the patient, and willingness for systemic therapy by the patients. Thus, ability to receive adjuvant chemotherapy was more closely equated to return-to-intended-oncological therapy (RIOT). Further surveillance of patients was as per the National Comprehensive Cancer Network (NCCN) guidelines, and CEA is repeated every 3 months and CECT of thorax, abdomen, and pelvis and is done every 6 months for the first two years. Then, CEA was performed every 6 months and CECT once in every year for the first 5 years [18, 19].

### Variables

Demographic variables, pathological characteristics of the primary tumour, type of metastasis, size, number and histopathology of liver metastasis, clinical risk score, neo-adjuvant systemic therapy offered, type of liver resection performed, adjunct use of liver directed therapies, patterns of recurrence and duration to develop recurrence and post-recurrence survival and therapeutic options offered were all the variables collected as a part of this study.

### Outcomes

Primary end-point of the study was site of first recurrence, categorised as either hepatic, extrahepatic, or both. The liver recurrences were further classified marginal (≤ 1 cm from resection bed) or extramarginal [20]. The secondary end-points were recurrence-free survival (RFS) and overall survival (OS) after curative CRLM resection. RFS was measured from the time of resection of liver metastases until recurrence or the last follow-up whilst OS was defined from the time of resection of liver metastases until death or the last follow-up. Liver relapse-free survival (LRFS) was considered till the appearance of liver recurrence.

### Statistical Analysis

Categorical data were expressed as frequencies and percentages, whereas continuous data were expressed as median and interquartile range (IQR). Survival curves were plotted using the Kaplan–Meier method. Follow-up duration was calculated using the reverse Kaplan–Meier method. Multivariable Cox regression analysis with backward elimination of variables with an exit level alpha of 0.2 was used to determine the factors that predicted the various relapse patterns. This gradual elimination of variables from higher *p*-values resulted in the transformation of a saturated regression model to a reduced model with important variables that allowed the best fit with the least possible number of variables from the data. All statistical analyses were conducted using SPSS v25 (IBM).

### Ethics

Data collection was in accordance with the Declaration of Helsinki [21]. As per institutional protocol, a formal board review was not taken for a retrospective analysis with anonymized data and absence of patient contact.

##   Results

Two hundred and fifty-eight CRLM patients treated with curative liver resection were analysed as a part of our study. The study population had a similar proportion of synchronous (144; 56%) and metachronous (114; 43%) CRLM. The median number of liver metastasis was two (Interquartile range: 1–4) with a median tumour size of 3.5 cm (IQR: 2–5 cm). A total of 159 (62%) patients received at least four cycles of neoadjuvant chemotherapy. A major hepatectomy (≥ 3 segments) was performed in 109 (45.7%) patients with unplanned positive resections margins (R +) in 36 (14%) patients. A total of 216 (84%) patients received the planned adjuvant therapy. The remaining patient characteristics are elucidated in Table 1 whilst the type of resections and therapeutic adjuncts are shown in Table 2.

**Table 1:** Patient characteristics

Characteristic	*N* = 258
Age	53 (42, 61)
Sex	
Male	165 (64%)
Female	93 (36%)
Type of metastasis	
Synchronous	144 (56%)
Metachronous	114 (44%)
Primary	
Colon	122 (47%)
Rectal	136 (53%)
Resection status of primary	
R0	258 (100%)
T stage	
T1	2 (0.8%)
T2	31 (12%)
T3	150 (58%)
T4	75 (29%)
N stage	
N0	67 (26%)
N1	117 (45%)
N2	74 (29%)
Extrahepatic metastasis	33 (13%)
Site of extrahepatic metastasis	
Lung	13 (5.0%)
Bone	2 (0.8%)
None	225 (87%)
Peritoneum	10 (3.9%)
Extra-regional nodes	8 (3.1%)
Number of liver metastasis	2 (1–4)
Largest dimension of liver metastasis	3.50 (2.00, 5.50)
CEA	14 (4, 42)
Fong’s clinical risk score	
0	8 (3.1%)
1	61 (24%)
2	71 (28%)
3	92 (36%)
4	23 (8.9%)
5	3 (1.2%)
Neoadjuvant chemotherapy	159 (62%)
Biologicals (*n* = 159)	
None	137 (86%)
Anti-EGFR	2 (1.3%)
Anti-VEGF	20 (13%)
Number of chemotherapy cycles (*n* = 159)	4.0 (4.0, 6.0)
Histologic subtype	
Well/moderately differentiated	227 (88%)
Poorly differentiated	31 (12%)
Lymphovascular invasion (*n* = 258)	24 (10%)

**Table 2:** Types of resections and therapeutic adjuncts

Characteristic	*N* = 258
Surgery performed	
Right hepatectomy	52 (20%)
Trisectionectomy	5 (1.9%)
Left hepatectomy	18 (7.0%)
Segmentectomy	8 (3.1%)
Left lateral sectionectomy	48 (19%)
Central hepatectomy	4 (1.6%)
Posterior sectionectomy	18 (7.0%)
Surgery with additional wedge resections	105 (41%)
Type of resection	
Anatomical	114 (44%)
Non-anatomical	121 (47%)
Anatomical & non-anatomical	23 (8.9%)
Number of segments resected	2 (1 – 4)
Major hepatectomy (≥ 3 segments)	109 (45.7%)
Simultaneous colorectal operation	92 (36%)
Significant complications (CD ≥ IIIA)	39 (15%)
Involved margins	36 (14%)
Margin width (mm)	10 (2, 20)
Adjuvant liver directed therapy (for smaller lesions)	43 (17%)
Type of liver directed therapy (*n* = 43)	
Trans-arterial chemoembolisation (TACE)	4 (9.3%)
Radio-frequency ablation (RFA)	27 (63%)
Hepatic artery infusion chemotherapy (HAIC)	9 (21%)
RFA + TACE	3 (7.0%)
Adjuvant systemic therapy	216 (84%)

The recurrence patterns identified are elaborated in Table 3. One hundred and fifty-six patients (156; 60%) relapsed after curative CRLM resection of which the majority were extra-hepatic distant recurrences (120; 47%) followed by liver relapses (91; 35%). One-hundred and five patients were recurrence-free, 33 (21.1%) had relapses in the liver, 62 (24.03%) had extrahepatic recurrences, and 58 (22.48%) had both, hepatic and extrahepatic, relapses. Isolated marginal liver relapses defined as those occurring within 1 cm of the liver resection margin were seen in only 7 (9.89%) patients out of the 91 liver recurrences. Extra-hepatic recurrences were observed in the lungs (66; 55%) followed by lymph nodes (22; 18%) and peritoneum (22; 18%).

**Table 3:** Relapse and post-recurrence outcomes:

Characteristic	*N* = 258
Recurrences	156 (60%)
Liver recurrence	91 (35%)
Marginal	7 (2.7%)
Liver recurrence outside marginal	84 (32.6%)
Extrahepatic recurrence	120 (47%)
Extrahepatic sites of relapse (*n* = 120)	
Lung	66 (55%)
Nodal	22 (18%)
Peritoneal	22 (18%)
Others	9 (7.6%)
Colorectal local recurrence	18 (7.0%)
Post-relapse treatment (*n* = 156)	
Systemic	74 (47%)
Liver directed	5 (3.2%)
Both	62 (40%)
None	15 (9.6%)
Liver directed therapy at recurrence (*n* = 91)	40 (45%)

**Fig. 1:**
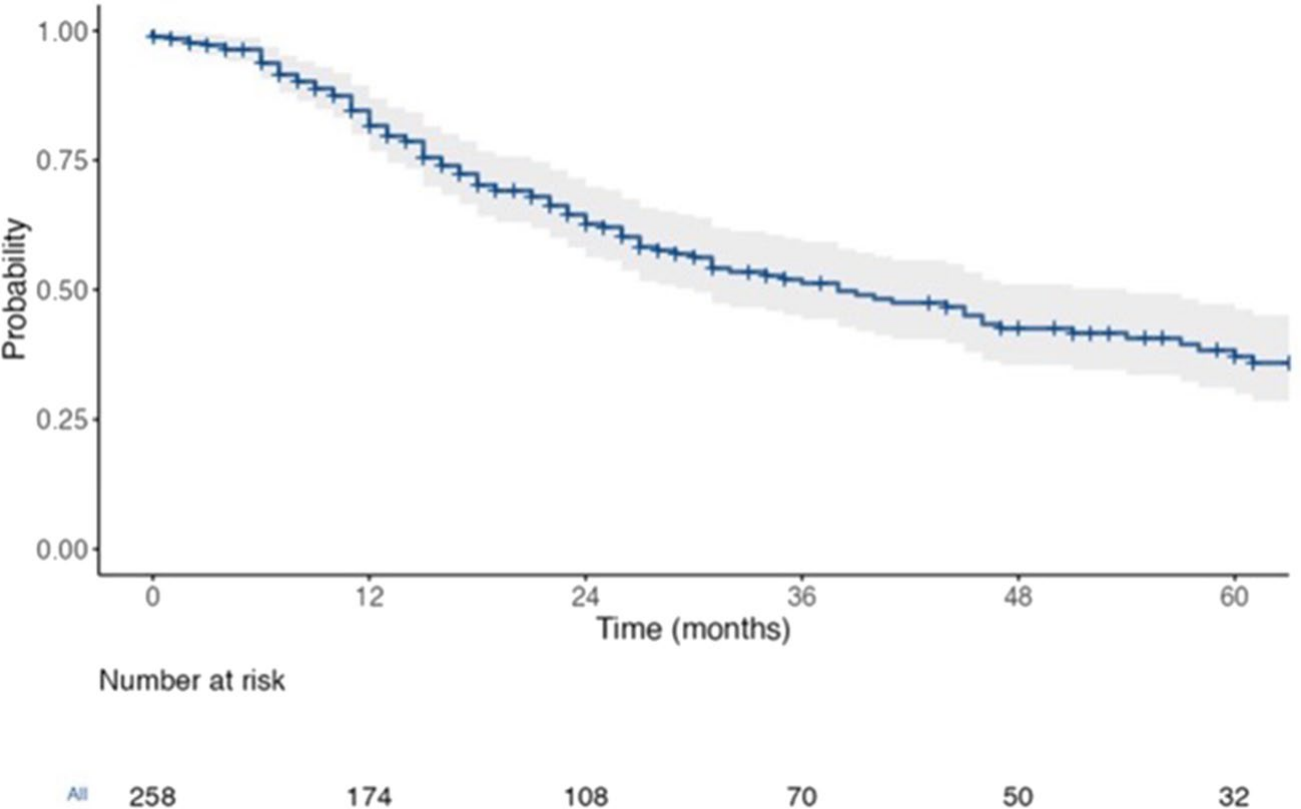
The OS of patients

**Fig. 2:**
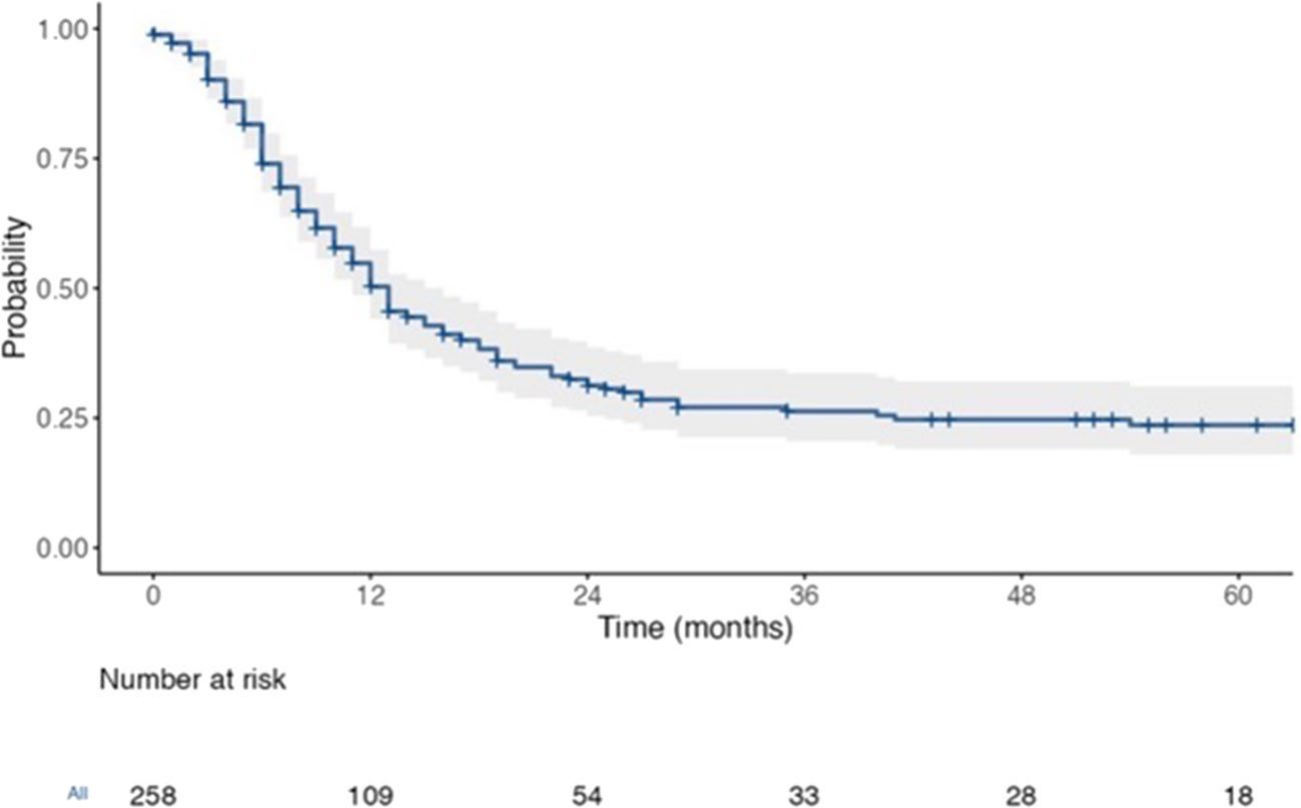
The OS of patients

At a median follow-up of 43 months (range 30–51 months, 95% CI), the OS of patients with resected CRLM was 38 months (30–54), the RFS was 13 months (11–16), and the 3-year LRFS was 54.4% (44.9–60.6) (Figs. 1 and 2). Amongst extra-hepatic sites, peritoneal recurrences (16.65 months, HR 1.53, *p* = 0.137) had worse survival compared to lung (19.31 months, HR 0.88, *p* = 0.553) and nodal (24.57 months, HR 0.80, *p* = 0.381) disease. The worst prognosis was seen with brain (3.2 months, HR 4.11, *p* = 0.05) and bone metastases (1.2 months, HR 4.43, *p* = 0.002), respectively, as they were often associated with other distant sites.

Size of liver metastases > 5 cm (HR 2.06 (1.34–3.17)), involved surgical margins (HR 2.16 (1.27–3.68)), omission of adjuvant chemotherapy (HR 1.89 (1.07–3.35)) were predictors of worse liver specific relapse-free survival (Table 4). Extrahepatic relapses were predicted by node positivity of primary (HR 1.61 (1.02–2.56), *p* < 0.041), presence of baseline extra-hepatic metastases (HR 0.30 (0.18–0.51), *p* < 0.01), size of liver metastases > 5 cm (HR 2.02 (1.37–2.99), *p* < 0.001), poorly differentiated histology (HR 2.25 (1.28–3.49), *p* = 0.043), presence of lymphovascular invasion (HR 2.25 (1.28–3.94), *p* = 0.005), and lack of adjuvant chemotherapy (HR 2.15 (1.28–3.61), *p* = 0.004) (Table 4). Timing and number of metastases or the use of preoperative chemotherapy did not influence survival outcomes. Factors that significantly affected overall survival were node positivity of the colorectal primary (HR 1.75 (1.09–2.80, *p* = 0.020)), size of the metastasis > 5 cm (HR 1.98 (1.33–2.94, *p* = 0.001), poorly differentiated histology (HR 4.12 (2.47–6.86, *p* < 0.001), presence of extrahepatic metastasis at baseline (HR 0.41 (0.25–0.69, *p* = 0.001), and ability to receive adjuvant chemotherapy (HR 2.66 (1.59–4.45, *p* < 0.001) (Table 5).

**Table 4:** Factors predicting liver recurrence and extra-hepatic recurrence

Variable		Liver recurrence HR (univariable)	Liver recurrence HR (multivariable)	Extra-hepatic HR (univariable)	Extra-hepatic HR (multivariable)
Timing of metastases	Synchronous	–	–	–	
	Metachronous	0.72 (0.47–1.11, *p* = 0.140)	0.81 (0.53–1.23, *p* = 0.319)	0.68 (0.47–1.00, *p* = 0.048)	
Node status of primary	Negative	–	–	–	–
	Positive	1.03 (0.63–1.67, *p* = 0.911)	1.08 (0.67–1.72, *p* = 0.762)	1.60 (1.01–2.53, *p* = 0.045)	1.61 (1.02–2.56, *p* = 0.041)
Baseline extrahepatic metastasis	Present	–	–	–	–
	Absent	0.93 (0.46–1.85, *p* = 0.826)	0.71 (0.37–1.36, *p* = 0.303)	0.40 (0.25–0.65, *p* < 0.001)	0.30 (0.18–0.51, *p* < 0.001)
Number of liver metastases	Single	–		–	–
	Multiple	1.19 (0.77–1.85, *p* = 0.431)		1.17 (0.80–1.71, *p* = 0.406)	
Size of liver metastases	< 5 cm	–	–	–	–
	≥ 5 cm	1.67 (1.09–2.57, *p* = 0.019)	2.06 (1.34–3.17, *p* = 0.001)	1.55 (1.07–2.24, *p* = 0.022)	2.02 (1.37–2.99, *p* < 0.001)
Preoperative CEA	< 200	–		–	
	≥ 200	1.24 (0.57–2.69, *p* = 0.584)		1.35 (0.71–2.59, *p* = 0.364)	
Neoadjuvant chemotherapy	No	–		–	
	Yes	1.07 (0.69–1.65, *p* = 0.757)		1.33 (0.91–1.93, *p* = 0.144)	
Pathology	Well/moderately differentiated	–	–	–	–
	Poorly differentiated	1.42 (0.73–2.76, *p* = 0.303)	1.37 (0.72–2.59, *p* = 0.338)	1.52 (0.87–2.67, *p* = 0.141)	1.81 (1.02–3.23, *p* = 0.043)
Lymphovascular invasion	No	–		–	**–**
	Yes	0.94 (0.43–2.05, *p* = 0.881)		1.92 (1.11–3.30, *p* = 0.019)	2.25 (1.28–3.94, *p* = 0.005)
Surgical margins	Free	–	–	–	
	Involved	1.90 (1.10–3.29, *p* = 0.021)	2.16 (1.27 3.68, *p* = 0.004)	0.84 (0.47–1.50, *p* = 0.552)	
Adjuvant chemotherapy		–	–	–	**−**
	None	1.79 (1.02–3.13, *p* = 0.042)	1.89 (1.07–3.35, *p* = 0.028)	1.66 (1.00–2.75, *p* = 0.049)	2.15 (1.28–3.61, *p* = 0.004)

**Table 5:** Factors predicting overall survival

Variable		Overall survival HR (univariable)	Overall survival HR (multivariable)
Timing of metastases	Synchronous	–	–
	Metachronous	0.78 (0.53–1.16, *p* = 0.218)	
Node status of primary	Negative	–	–
	Positive	1.43 (0.89–2.29, *p* = 0.141)	1.75 (1.09–2.80, *p* = 0.020)
Baseline extrahepatic metastasis	Present	–	–
	Absent	0.48 (0.28–0.83, *p* = 0.008)	0.41 (0.25–0.69, *p* = 0.001)
Number of liver metastases	Single	–	–
	Multiple	1.07 (0.72–1.59, *p* = 0.724)	
Size of liver metastases	< 5 cm	–	–
	≥ 5 cm	1.41 (0.96–2.08, *p* = 0.084)	1.98 (1.33–2.94, *p* = 0.001)
Preoperative CEA	< 200	–	–
	≥ 200	1.02 (0.49–2.09, *p* = 0.967)	
Neoadjuvant chemotherapy	No	–	–
	Yes	1.48 (0.99–2.21, *p* = 0.057)	
Pathology	Well/moderately differentiated	–	–
	Poorly differentiated	3.16 (1.88–5.29, *p* < 0.001)	4.12 (2.47–6.86, *p* < 0.001)
Lymphovascular invasion	No	–	–
	Yes	1.35 (0.70–2.61, *p* = 0.365)	
Margins	Free	–	–
	Involved	1.32 (0.76–2.28, *p* = 0.325)	1.40 (0.82–2.38, *p* = 0.212)
Adjuvant	Chemotherapy	–	–
	None	1.96 (1.19–3.24, *p* = 0.008)	2.66 (1.59–4.45, *p* < 0.001)

## Discussion

Our study of 258 resected colorectal liver metastasis was conducted with the main aim to identify which patients recur in liver or extrahepatic sites after curative liver resection and delineate the factors which affect this pattern of recurrence. Our study analysed data on recurrence patterns and found that majority of the relapses were within extrahepatic sites followed by the liver. However, marginal recurrences around the resection bed were minimal. Tumour size and the inability to receive adjuvant therapy were common predictors of hepatic and extra-hepatic recurrences whilst extrahepatic relapses were additionally predicted by nodal stage, extrahepatic disease at baseline, poorly differentiated histology, and lymphovascular invasion.

The prognostic implications of margins in CRLM resections have been a subject of long debate. Whilst R0 resections with at least 1 mm of clear margin should always be the goal, a microscopically positive surgical margin or a clear margin of less than 1 mm (R1) on pathology is a grey area. Though previous studies showed no significant difference in median overall survival and recurrence-free survival amongst patients with R0 or R1 resection in high risk groups, overall R1 resections were associated with higher local failure [15, 22–24]. Our data reveals very few had marginal recurrences (7; 2.7%) even though the margin positivity rate was 14%. Since, R0 resections were always the goal and marginal recurrences were so few in number, increasing the width of resection may not be the solution and the failure to achieve clear margins may only reflect poor tumour biology. Caution should be exercised in the interpreting the results since no patient in the present study had planned positive margins, and we do not suggest that an R1 resection is acceptable based on the present study. The practice of intentional R1 vascular margins proposed by Vigano et al. which were reported to achieve outcomes equivalent to true R0 resections is emerging and accepted though we have not applied it in our patients [17]. There is some evidence of correlation between the margin status (R0 vs R1) and OS based on tumour biology. The type of margin status in wtKRAS tumours was shown to affect both OS and liver recurrence free survival whilst it was minor in mKRAS tumours; however, more data would be required to suggest any practice modification [14].

The results from our study suggest that the ability to receive adjuvant chemotherapy after curative liver resection positively impacted survival. Thus, patients with a good performance status who recovered with negligible surgical morbidity did better. Perioperative chemotherapy is routinely recommended by current National Comprehensive Cancer Network (NCCN) guidelines based on retrospective data [25–27]. Buisman et al. showed in a competing risk analysis that perioperative systemic chemotherapy decreases extrahepatic recurrences in high risk patients [27]. Careful interpretation is necessary since randomised trials have not proven overall survival benefit, even though improvements in RFS have been reported [28, 29]. Current practice for adjuvant therapy in CRLM is a controversy world over in view of the conflicting results in phase III trials whose primary end point was RFS and hence were probably underpowered to detect overall survival benefit. Bearing in mind the systemic toxicity, lack of strong evidence to correlate RFS to OS, and lack of data on patient reported quality of life outcomes, we accept further evidence is necessary [14, 30]. We recommend future analysis of available data with risk stratification for recurrence based on other risk factors and research to identify better prognostic and predictive markers.

Older studies evaluating various prognostic nomograms suggest size and number of liver metastasis, lymph node status in primary tumour and pre-operative CEA levels to be the most commonly included factors [31]. Our results indicate that tumour size is of the most prognostic value amongst different components of Fong’s clinical risk score This is following multiple recent studies which showed that even with the inclusion of RAS status improved predictive power, tumour size and number are still the most significant variables [28, 32–34]. Thus, it is possible that different components of the risk score have differential weights, and it may be prudent to revisit the scoring system.

A frequently debated contraindication for local treatment of CRLM is the simultaneous presence of extrahepatic disease. Several retrospective studies support CRLM resection with concurrent extrahepatic disease confined to a single organ in highly selected patients [35–37]. Our study analysis had a higher risk of disease recurrence in the presence of baseline extra-hepatic metastasis, which is expected.

Even though the size of metastasis and inability to receive adjuvant chemotherapy are the most consistent predictors of recurrence of any recurrence, distant recurrences were additionally predicted by node-positive primary cancer, poorly differentiated histology, extrahepatic metastasis, and lymphovascular invasion. Thus, the presence of these features can guide the adjuvant therapy in patients. A tumour with liver only metastasis with a size > 5 cm without other adverse features should probably be subjected to additional liver-directed therapy in the form of hepatic artery infusional chemotherapy (HAIC) during systemic therapy. Finally, given the ambiguity surrounding the role of perioperative chemotherapy in limited, resectable CRLM, systemic therapy may be avoided in those without any of the risk factors.

Application of liver-directed therapies as adjunct in the treatment of CRLM has long been commonplace at our institute [38]. Augmentation of liver directed therapies like hepatic artery infusion chemotherapy (HAIC) or trans-arterial chemoembolization (TACE) has been applied in the management of initially unresectable and heavily treated CRLM all over the world now. The evidence for role of HAIC as adjunct to adjuvant systemic chemotherapy is limited but promises improvement in overall survival [39, 40]. This evidence is further strengthened by findings from the PACHA-01 trial where adjuvant HAIC has a role in improving liver specific recurrence free survival in high risk patients [41]. At our institute, HAIC is offered to select patients as adjuvant therapy with more than 3 liver metastasis, bilobar lesions or > 5 cm, or for unresectable disease that has failed 2 lines of chemotherapy with or without TACE [42].

Overall, elucidation of different patterns of recurrence and the factors affecting the pattern can be refined with further research to identify patients with individualised risk. This will allow formulation of more reliable precision medicine strategies to allow neoadjuvant and adjuvant therapies to be tailored for each risk group. Future trials in hepatic arterial infusion therapy can include patients with higher risk of a liver recurrence, resulting in greater efficacy. More analysis of real-world data on management of colorectal liver metastases is necessary to derive conclusions.

Our study has a few limitations as well. The relatively small sample and retrospective study design from a single institution is subject to selection bias and limited external validity. In addition, the extended period included in the study may combine management strategies from different eras and evolving systemic therapy regimens. Addition of a contrast enhanced MRI of the liver to CT has shown up to 30% change in treatment plan. Since the use of MRI was not universal in our patients, the same can be a potential limitation as well [43]. Influence of histological growth patterns (desmoplastic vs non-desmoplastic) and molecular biomarkers like RAS alterations, BRAF and TP53 mutations, microsatellite instability and subsequent mismatch repair gene deficiency and circulating tumour cells were not included in analysis and is a strong limitation to our data [32, 33, 44–48].

## Conclusion

Amongst resected CRLM, the majority of relapses occurred at extrahepatic sites followed by that in the liver. However, isolated marginal recurrences were very few. Most consistent predictors of recurrence were size and the inability to deliver adjuvant therapy. Extrahepatic relapses were additionally predicted by nodal stage, baseline extrahepatic disease, and histologic differentiation. With further research, tailored adjuvant therapy might improve patient outcomes after liver metastasectomy in colorectal cancers.

## Data Availability

No datasets were generated or analysed during the current study.

## Abbreviations

CRLM: Colorectal liver metastasis
CEA: Carcinoma embryonic antigen
MRI: Magnetic Resonance Imaging
PET: Positron Emission Tomography
MDT: Multi-disciplinary team
RIOT: Return-to-intended-oncological therapy
NCCN: National Comprehensive Cancer Network
RFS: Relapse-free survival
OS: Overall survival
LRFS: Liver relapse-free survival
HAIC: Hepatic artery infusion chemotherapy
RFA: Radio-frequency ablation
TACE: Trans-arterial chemoembolization
CD: Clavien Dindo
RAS gene: Rat sarcoma gene
BRAF gene: V-Raf murine sarcoma viral oncogene homolog B1
